# Supplementary material for: Causal Relationship between Aging and Anorexia Nervosa: A White-Matter-Microstructure-Mediated Mendelian Randomization Analysis
Source: Biomedicines. 2024 Aug 16;12(8):1874. doi: 10.3390/biomedicines12081874 (PMC11351342; doi:10.3390/biomedicines12081874)
Supplement: Supplementary file 1 [file biomedicines-12-01874-s001.zip › Supplementary Figure/Figure S8/Leaveoneout plot of MR analysis for TL to ubm-b-1472.pdf]

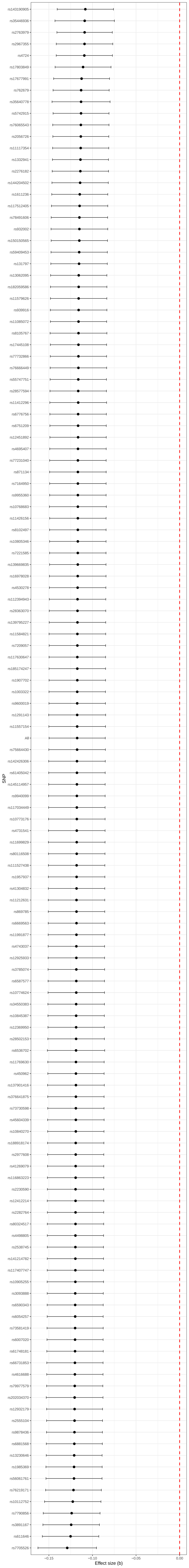
rs143190905
rs35446936
rs2763979
rs2967355
rs4724
rs17803849
rs17677991
rs762679
rs35640778
rs5742915
rs76065543
rs2056726
rs11117354
rs1332941
rs2276182
rs144204502
rs1611236
rs117512405
rs78491606
rs932002
rs150150565
rs59409453
rs131797
rs13062095
rs182059586
rs11579626
rs939916
rs11085072
rs8105767
rs17445108
rs77732866
rs76666449
rs55747751
rs28577594
rs11412296
rs6776756
rs6751209
rs12451892
rs4695407
rs77231040
rs871134
rs7164950
rs9955360
rs10768683
rs11426156
rs8102497
rs10805346
rs7221585
rs139669835
rs16978028
rs4530278
rs112394943
rs28363070
rs139795227
rs11584821
rs7209057
rs117630647
rs185174247
rs1907702
rs1003322
rs9600019
rs1291143
rs11557154
All
rs75664430
rs142426306
rs61405042
rs145114957
rs9940099
rs117034449
rs10773176
rs4731541
rs11699829
rs80116508
rs111527438
rs1957937
rs41304832
rs11212631
rs869785
rs6669563
rs11991877
rs4743037
rs12925933
rs3785074
rs6587577
rs10774624
rs34550383
rs10845387
rs12369950
rs28502153
rs6536702
rs11769630
rs450962
rs137901416
rs376641875
rs73730598
rs45604339
rs10840270
rs188918174
rs2977608
rs41269079
rs116863223
rs2230590
rs12412214
rs2282764
rs80324517
rs4498805
rs2538745
rs141214782
rs117407747
rs10905255
rs3093888
rs6590343
rs6054257
rs73581419
rs6007020
rs61748181
rs66731853
rs4616688
rs79977579
rs202034370
rs12932179
rs2555104
rs9878436
rs6881568
rs13230646
rs1985369
rs56061761
rs76219171
rs10112752
rs7790856
rs3891167
rs611646
rs7705526
SNP
−0.15
−0.10
−0.05
0.00
Effect size (b)
